# Incision capsular bag herniation during subluxated lens surgery: mechanisms, management, and prevention

**DOI:** 10.3389/fmed.2025.1647413

**Published:** 2025-08-25

**Authors:** Shuang Ni, Danni Lyu, Jianxia Fang, Wen Xu

**Affiliations:** ^1^Eye Center, The Second Affiliated Hospital, School of Medicine, Zhejiang University, Hangzhou, China; ^2^Zhejiang Provincial Key Lab of Ophthalmology, The Second Affiliated Hospital, School of Medicine, Zhejiang University, Hangzhou, China

**Keywords:** capsular bag herniation, lens subluxation, zonular dialysis, intraocular pressure, intraoperative complications

## Abstract

**Purpose:**

To investigate the mechanism, intraoperative characteristics, management, and prevention of incision capsular bag herniation (ICBH), a previously unreported complication during cataract surgery in eyes with lens subluxation.

**Methods:**

A retrospective observational case series was conducted on five male patients who developed ICBH during phacoemulsification with intraocular lens (IOL) implantation between January 2019 and December 2024. Among 867 subluxated-lens surgeries performed during this period, the estimated incidence of ICBH was 0.58%. Clinical data was reviewed to identify predisposing factors and outcomes. Each case was managed through a stepwise intraoperative process consisting of IOP reduction, capsular decompression, capsular bag repositioning, and anterior vitrectomy.

**Results:**

ICBH occurred adjacent to the main corneal incision near areas of 1–2 quadrants of zonular dialysis. Triggers included IOL injector insertion (2 cases), ophthalmic viscosurgical device (OVD) injection (1 case), and aspiration tip withdrawal (2 cases). Contributing factors included elevated IOP and sudden decompression. The herniated tissue comprised an OVD-distended capsular bag with vitreous incarceration. The stepwise protocol led to successful capsular repositioning and in-the-bag IOL implantation in all cases. Best-corrected visual acuity improved from a preoperative range of 1.3 to 0.3 logMAR (median, 0.5) to a postoperative range of 0.7 to 0.0 logMAR (median, 0.1). No IOL decentration occurred. One case each of cystoid macular edema and choroidal detachment was noted.

**Conclusion:**

ICBH results from zonular weakness, incision location, IOP fluctuation, OVD dynamics, and capsular biomechanics. With appropriate intraoperative management, favorable outcomes are achievable. Prevention includes zonular assessment, incision planning, and pressure control. Further studies are needed.

## Introduction

Lens subluxation, characterized by partial zonular dehiscence or dysfunction resulting in displacement of the crystalline lens from its anatomical position, is frequently associated with traumatic, congenital or degenerative conditions (1). Surgical intervention is often necessary when significant visual impairment, secondary glaucoma, or uveitis occurs (2, 3). However, these procedures are notably more complex and have higher rates of intraoperative complications compared to standard cataract surgery, primarily due to challenges such as zonular weakness, uneven capsular tension, vitreous prolapse, and limited surgical space (4). Tears of the anterior and posterior capsules, along with the progressive extension of zonular dehiscence, are common complications associated with capsular bag involvement during subluxation surgery (1, 5, 6). In addition to these complications, a rare intraoperative event is the herniation of the capsular bag through the main corneal incision, followed by irreversible incarceration and the formation of a non-reducible capsular hernia. To the best of our knowledge, this phenomenon has not been previously reported in the literature. This study is the first to systematically investigate its occurrence, mechanisms, and management in the context of subluxated lens surgery. We refer to this complication as incision capsular bag herniation (ICBH) to distinguish it from other known intraoperative entities such as iris or vitreous prolapse. ICBH is characterized by the outward ballooning of the capsular bag through the incision without frank rupture, requiring more precise surgical manipulation. Improper management of this complication can lead to capsular rupture, to hinder in-the-bag intraocular lens (IOL) implantation. Conversely, timely and appropriate intervention can preserve capsular integrity and facilitate successful IOL placement. ICBH remains insufficiently understood in terms of its underlying mechanisms. This retrospective study analyzes five cases of ICBH managed at our institution over 6 years, with the goal of elucidating its pathogenesis, refining surgical management strategies, and proposing preventive measures to improve clinical outcomes.

## Methods

This retrospective observational case series included five cases with lens subluxation underwent phacoemulsification and IOL implantation at the Eye Center of the Second Affiliated Hospital of Zhejiang University School of Medicine from January 2019 to December 2024. These cases were identified from 867 subluxated-lens surgeries performed by the same surgical team, resulting in an estimated ICBH incidence of 0.58%. All included cases experienced intraoperative ICBH. Surgeries were conducted by experienced cataract surgeons (W.X. and S.N.), and each patient underwent a postoperative follow-up period of at least 3 months.

The standardized surgical protocol involved: creation of a 0.8-mm side-port corneal incision at the 2 o’clock position and a 2.0-mm clear corneal main incision at the 10 o’clock position; stabilization of the anterior chamber with an ophthalmic viscosurgical device (OVD) (Hyaluronate Sodium, Shandong Bausch & Lomb Freda Pharmaceutical Co., Ltd., Shangdong, China), with additional iris hooks required in Case 3 for posterior synechiolysis; and continuous curvilinear capsulorhexis (CCC) with a target diameter of 5.5 mm performed using capsulorhexis forceps. Capsular tension ring (CTR) implantation was performed in four cases, whereas it was omitted in Case 3 due to sufficient zonular stability. Phacoemulsification was performed using the Stellaris Phacoemulsification System (Bausch + Lomb, Rochester, NY, United States) with a vacuum setting of 320 mmHg, ultrasound power of 70%, and bottle height of 90 cm.

**Figure 1:**
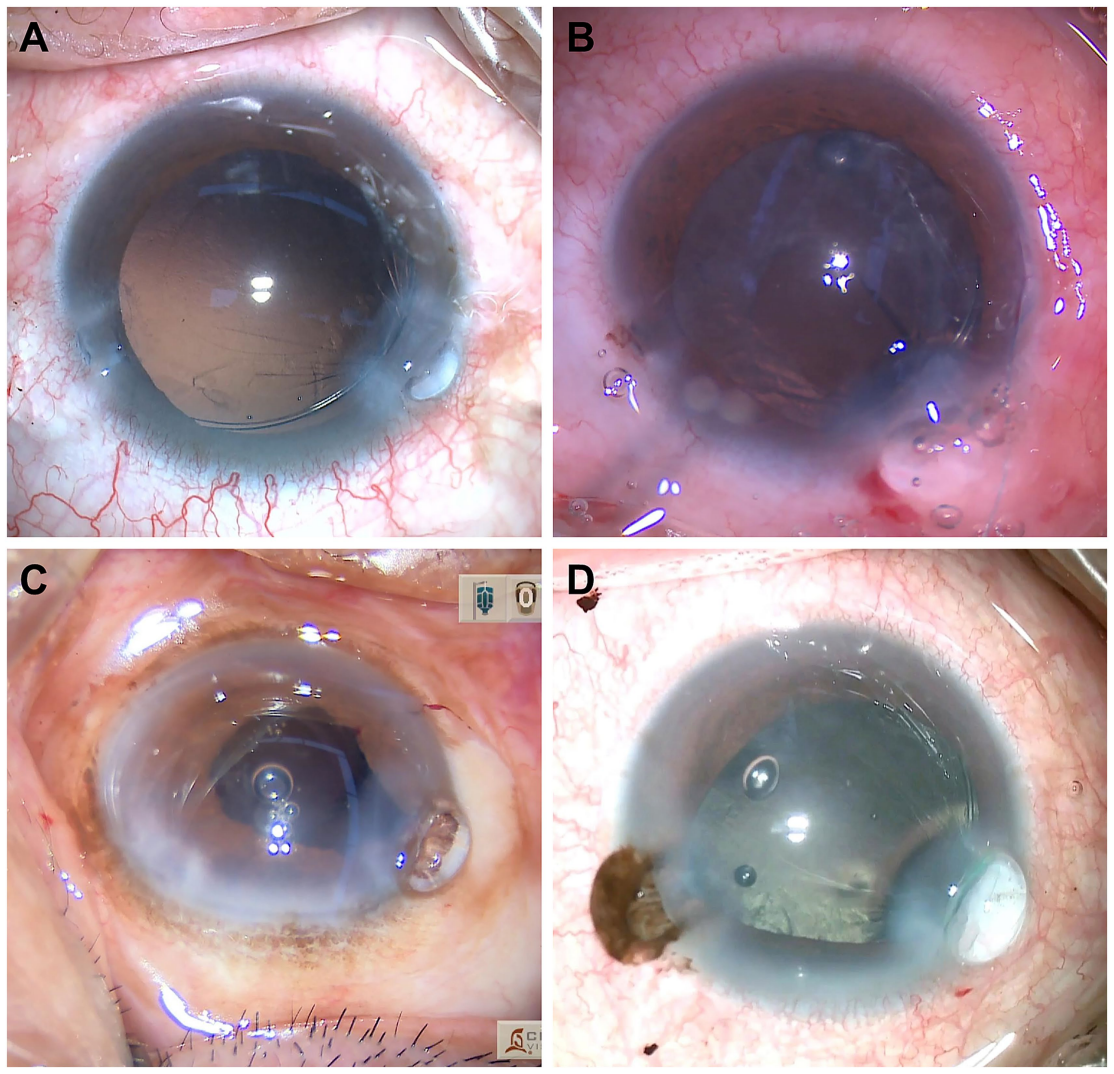
Intraoperative incision capsular bag herniation (ICBH) in four cases. ICBH occurred at different intraoperative stages in subluxated lens surgery. **(A)** Case 1: ICBH observed during insertion of the IOL injector through the main incision. **(B)** Case 2: ICBH noted during supplemental injection of ophthalmic viscosurgical device (OVD) via the side-port. **(C)** Case 3: ICBH developed during withdrawal of the irrigation/aspiration handpiece tip while removing residual cortex and OVD. **(D)** Case 4: ICBH developed during withdrawal of the irrigation/aspiration handpiece tip while removing residual cortex and OVD.

ICBH occurred at various stages of surgery: Cases 1 (Figure 1A) and Cases 5 (no intraoperative video recording) during insertion of the IOL injector through the main incision, Case 2 (Figure 1B) during supplemental OVD injection via the side port, Case 3 and Case 4 during withdrawal of the irrigation/aspiration (I/A) handpiece tip while removing residual cortical material and OVD (Figures 1C,D), with settings of 90 cm bottle height and 450 mmHg vacuum. In all cases, intraoperative ICBH occurred, characterized by capsular bag herniation through the main incision and distension by OVD, requiring manual repositioning.

**Figure 2:**
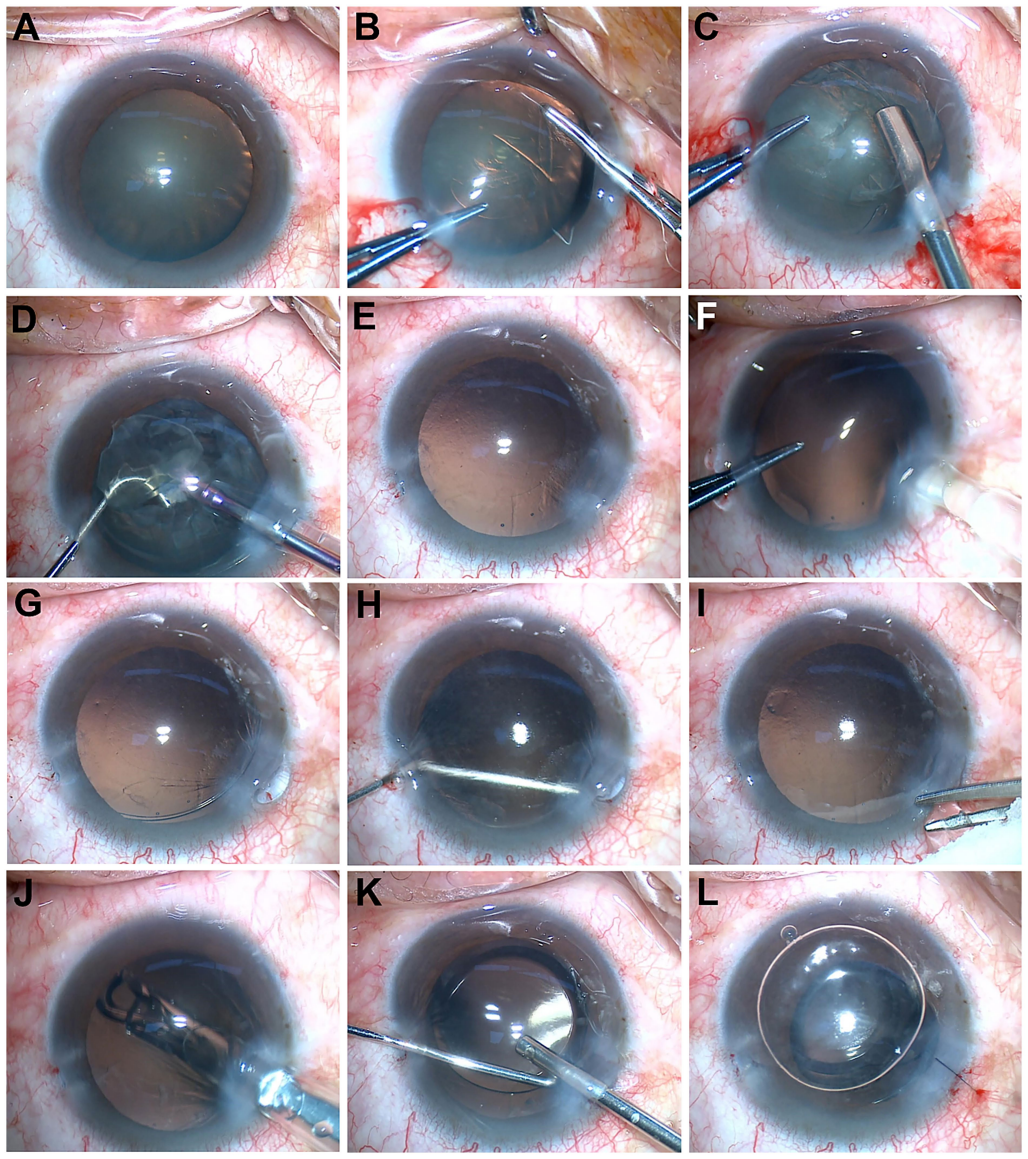
Representative intraoperative steps of surgical management illustrated using Case 1. **(A)** Preoperative view showing capsular folds at the superior equator, suggestive of zonular weakness. **(B)** Creation of a continuous curvilinear capsulorhexis. **(C)** Insertion of a capsular tension ring (CTR) between the capsular bag and cortical material. **(D)** Phacoemulsification of the lens nucleus and cortex. **(E)** Well-centered capsulorhexis margin following lens removal. **(F)** Contact between the intraocular lens (IOL) injector tip and the main corneal incision. **(G)** Partial herniation of the capsular bag through the main incision. **(H)** Repositioning of the herniated portion of the capsular bag using a blunt cannula via the side-port incision. **(I)** Anterior vitrectomy using a vitreous cutter to remove prolapsed vitreous strands and restore capsular bag centration. **(J)** IOL implantation with an injector. **(K)** Anterior pars plana vitrectomy to clear residual vitreous. **(L)** Closure of the main corneal incision with 10–0 nylon sutures, followed by air injection through the side port to reform the anterior chamber.

The standardized management approach consisted of: (1) releasing the posterior lip of the main incision by introducing a blunt cannula through the side port; (2) gently repositioning the herniated capsule; (3) implanting a foldable IOL; (4) anterior vitrectomy was performed when vitreous was observed prolapsing into the anterior chamber or when signs of vitreous traction were evident intraoperatively; and (5) closing the incision with 10–0 sutures and injecting disinfected air through the side port to reform the anterior chamber. Postoperative medical therapy comprised topical tobramycin-dexamethasone ointment, prednisolone acetate 1% four times daily, levofloxacin 0.5% four times daily, and diclofenac sodium 0.1% four times daily. (The detailed surgical procedure is illustrated in Figure 2 and Supplementary Video 1.)

Collected data included: demographic information (age, sex, systemic comorbidities, etiology of zonulopathy); Preoperative assessments included intraocular pressure (IOP), uncorrected and best-corrected visual acuity (UCVA and BCVA) measured using a standard LogMAR chart, anterior chamber depth assessed by ultrasound biomicroscopy, and the extent of zonular dialysis evaluated by slit-lamp examination after pharmacologic pupil dilation; intraoperative details (iris condition, CTR usage, and characteristics of capsular herniation); and postoperative outcomes (visual acuity, IOP and complications at 3 month). Given the limited sample size (*n* = 5), descriptive statistical methods were utilized. All statistical analyses were performed using SPSS (version 26.0; SPSS Inc., Chicago, IL, United States). Data were summarized using medians and ranges. This study protocol was approved by the Institutional Review Board of the Second Affiliated Hospital of Zhejiang University School of Medicine. All procedures adhered to the tenets of the Declaration of Helsinki. All clinical data were meticulously retrieved from the electronic medical record system, with the patient’s explicit written consent duly obtained.

## Results

This study included five male cases (Cases 1–5; Table 1) aged from 51 to 79 years (median, 66 years). Etiological analysis identified traumatic lens subluxation in four cases (80%; Cases 1, 2, 4, and 5), while one case (20%; Case 3) had nanophthalmos associated with retinitis pigmentosa, high hyperopia, and angle-closure glaucoma without preoperative lens displacement. Preoperative evaluation revealed zonular dialysis involving 1.5 quadrants in three cases (Cases 1, 4, and 5) and 2 quadrants in one case (Case 2). Visual acuity assessments showed preoperative visual acuity ranged from 2.0 to 1.0 logMAR for UCVA (median, 1.0 logMAR) and from 1.3 to 0.3 logMAR for BCVA (median, 0.5 logMAR). Anterior segment parameters included anterior chamber depth (ACD) ranging from 1.31 to 3.68 mm (median, 2.68 mm) and IOP between 11 and 19 mmHg (median, 15 mmHg).

**Table 1:** Baseline characteristics of cases with incision capsular bag herniation (ICBH).

Case	Age/Sex	Etiological factor	Subluxation extent	UCVA LogMAR	BCVA LogMAR	ACD (mm)	IOP (mmHg)	Comment
1	65/M	Blunt ocular trauma	Superior region, approximately 1.5 quadrants	1.0	0.3	1.88	16	None
2	51/M	Blunt ocular trauma	Superior region, approximately 2 quadrants	1.0	0.5	3.21	11	None
3	66/M	Nanophthalmos	Not detected preoperatively	2.0	1.3	1.31	19	Retinitis pigmentosa, high hyperopia, and angle-closure glaucoma
4	79/M	Blunt ocular trauma	Superior region, approximately 1.5 quadrants	1.0	0.5	3.68	15	None
5	66/M	Blunt ocular trauma	Superior region, approximately 1.5 quadrants	1.3	0.7	2.68	13	None

M, Male; UCVA, Uncorrected Visual Acuity; BCVA, Best-Corrected Visual Acuity; ACD, Anterior Chamber Depth; IOP, Intraocular Pressure.

Intraoperative findings (Table 2) confirmed zonular weakness in all cases, with dialysis involving approximately 1.5 quadrants (Cases 1, 4, and 5, 60%), 2 quadrants (Case 2, 20%), and 1 quadrant (Case 3, 20%). ICBH was consistently observed adjacent to the superior main corneal incision in all cases (100%). Surgical management included CTR implantation in four cases (Cases 1, 2, 4, and 5; 80%). Case 2 additionally required scleral suture fixation through a scleral reverse pocket due to greater zonular instability. Iris hooks were used exclusively in Case 3 (20%) to address posterior synechiae. The timing of ICBH varied: during IOL injector insertion (Cases 1 and 5, 40%), OVD injection (Cases 2, 20%), and withdrawal of the I/A handpiece tip (Case 3 and 4, 40%).

**Table 2:** Intraoperative characteristics and management of cases with incision capsular bag herniation (ICBH).

Case	Zonular abnormality	CTR timing	Capsule intact	Iris status	Herniation timing	Cortex present	Elevated IOP	OVD overfilling	Vitreous incarceration	Capsule repositioned	IOL implanted	Vitreous removed	Subluxation enlarged
1	Superior ~1.5 quadrants	After hydrodissection	Yes	Normal	Injector tip contact	No	Yes	Yes	Yes	Yes	Yes	Yes	No
2	Superior ~2 quadrants	After hydrodissection; scleral fixation	Yes	Normal	During cortex removal and OVD injection	Yes	Yes	Yes	Yes	Yes	Yes	Yes	No
3	Superior ~1 quadrant	Not implanted	Yes	Posterior synechiae; iris hooks	Post-IOL implantation; I/A tip withdrawal	Yes	Yes	Yes	Yes	Yes	Yes	Yes	Yes, intraoperative discovery
4	Superior ~1.5 quadrants	After hydrodissection	Yes	Iris flaccidity	Aspiration of residual cortex and OVD; upon I/A tip withdrawal	Yes	Yes	Yes	Yes	Yes	Yes	Yes	No
5	Superior ~1.5 quadrants	After hydrodissection	Yes	Normal	Injector tip contact	No	Yes	Yes	Yes	Yes	Yes	Yes	No

CTR, Capsular Tension Ring; OVD, Ophthalmic Viscosurgical Device; IOP, Intraocular Pressure; I/A, Irrigation/Aspiration.

Critical intraoperative findings included no nuclear fragment retention (0%), cortical remnants in three cases (Cases 2, 3, and 4, 60%), elevated intraocular pressure in all cases (100%), significant OVD retention in all cases (100%), and vitreous prolapse through the main incision in all cases (100%). Manual reduction of the herniated portion of the capsular bag and successful in-the-bag IOL implantation were achieved in all cases (100%), accompanied by routine and complete anterior vitrectomy. Progression of zonular dialysis was observed only in Case 3 (20%).

Postoperative follow-up (3 months; Table 3) revealed no significant IOL decentration in any case. Visual outcomes improved, with UCVA ranging from 1.30 to 0.00 logMAR (median, 0.2 logMAR) and BCVA from 0.7 to 0.0 logMAR (median, 0.1 logMAR). Stable IOP was maintained at 12 to18 mmHg (median, 15 mmHg). Transient corneal edema resolved completely within 2 weeks in all cases (100%). Complications included cystoid macular edema at 1 month in Case 2, and postoperative choroidal detachment in Case 3.

**Table 3:** Postoperative outcomes of cases with incision capsular bag herniation (ICBH).

Case	UCVA LogMAR	BCVA LogMAR	IOP (mmHg)	IOL decentration	Temporary corneal edema	Postoperative complications
1	0.2	0.1	13	no	Present	None
2	0.4	0.2	12	no	Present	Cystoid macular edema at 1 month
3	1.3	0.7	18	no	Present	Choroidal detachment at 1 month
4	0.2	0.1	16	no	Present	None
5	0.0	0.0	15	no	Present	None

UCVA, Uncorrected Visual Acuity; BCVA, Best-Corrected Visual Acuity; IOP, Intraocular Pressure; IOL, Intraocular Lens.

## Discussion

ICBH is a previously unreported complication observed during cataract surgery in eyes with lens subluxation. Despite its potential impact on surgical outcomes, the mechanisms, management approaches, and preventive strategies for ICBH have not been clearly defined in the existing ophthalmic literature. This study presents the first systematic description of ICBH as a distinct intraoperative event, based on a retrospective analysis of five cases, offering new insights into its recognition and handling.

**Figure 3:**
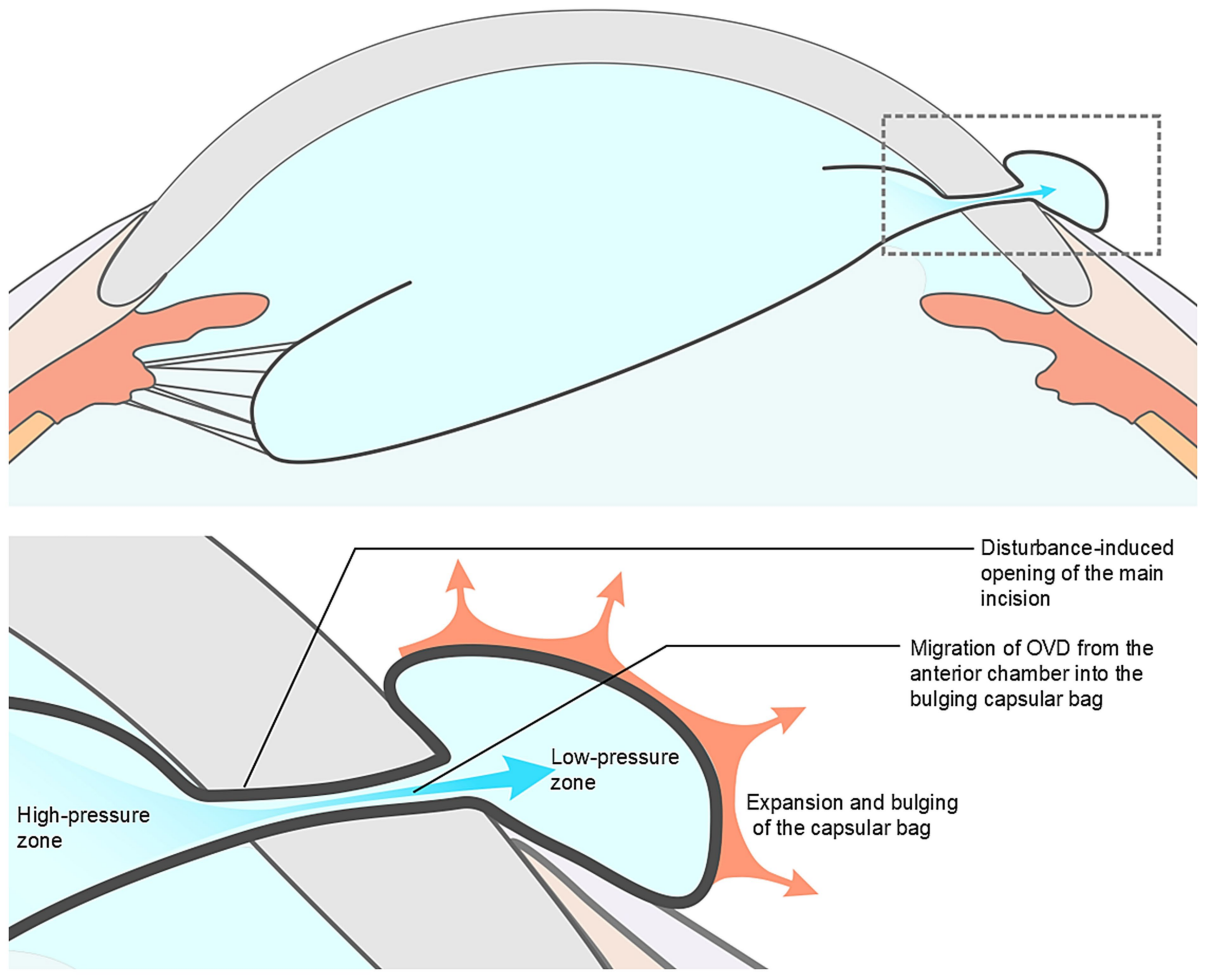
Proposed mechanism of incision capsular bag herniation (ICBH). Intraocular pressure within the anterior chamber exceeds that of the herniated portion of the capsular bag, resulting in ophthalmic viscosurgical device (OVD) inflow along the pressure gradient and further expansion of the herniated portion of the capsular bag.

**Figure 4:**
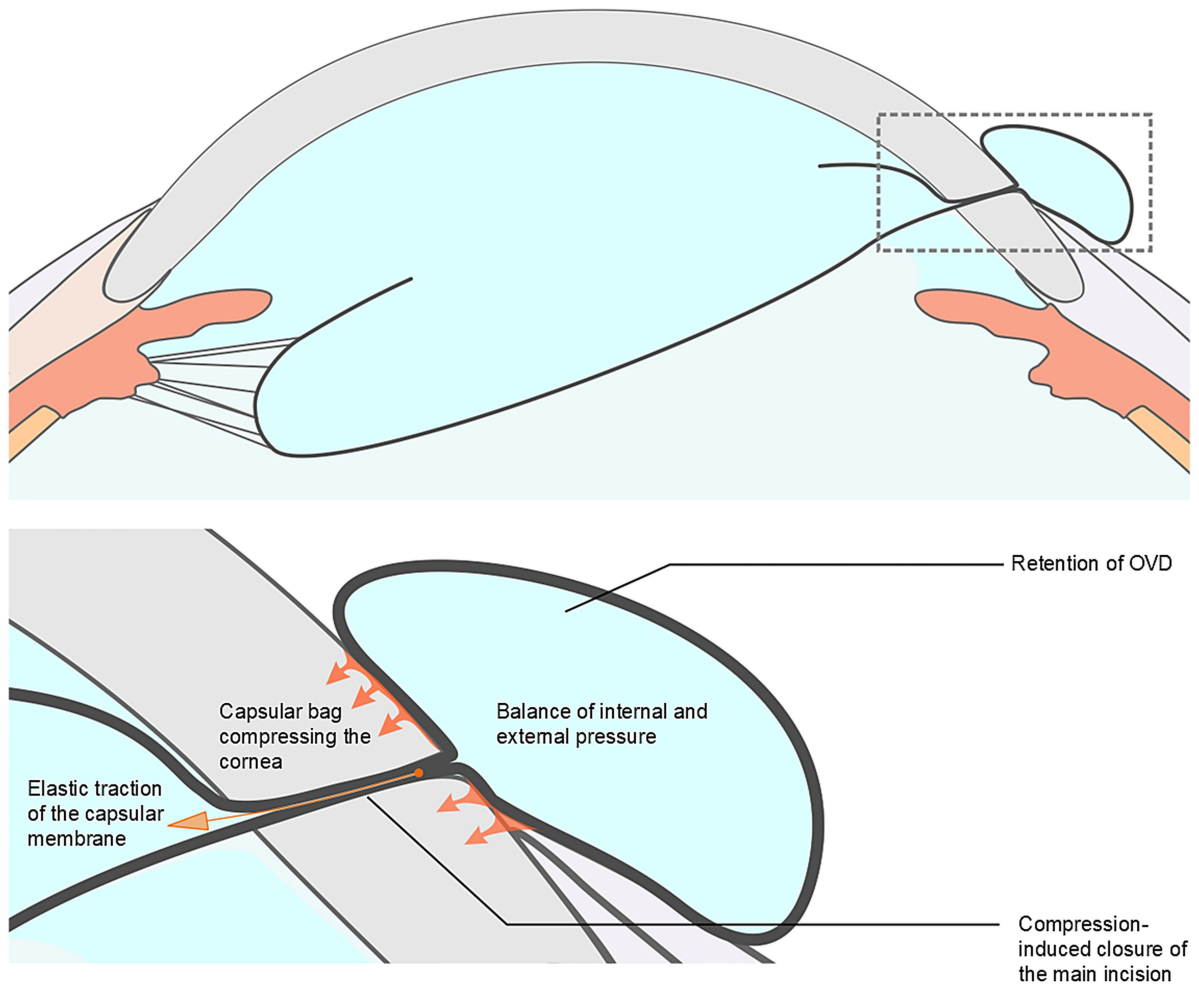
Proposed mechanism of difficult repositioning of capsular bag. Formation of an ophthalmic viscosurgical device (OVD)-distended, mushroom-shaped capsular protrusion, with elastic recoil constricting the incision edges and creating an irreducible incarcerated capsular hernia.

Our analysis suggests several possible mechanisms underlying ICBH: (1) Extensive zonular dialysis, which results in increased capsular mobility (7); (2) Superior zonular weakness adjacent to the main corneal incision, predisposing the capsule to herniate; (3) Elevated intraoperative IOP, resulting from excessive OVD injection or elevated irrigation pressure (8); (4) Sudden decompression of the anterior chamber due to compromised main incision integrity (instrument manipulation or inadequate incision construction), causing rapid outward fluid egress and subsequent capsular bag and vitreous prolapse through the incision; (5) The IOP within the anterior chamber exceeds the pressure inside the herniated portion of the capsular bag, leading to the flow of OVD into the herniated capsule along the pressure gradient, thereby causing further expansion of the herniated portion of the capsular bag (see Figure 3); and (6) Formation of a OVD-distended, mushroom-shaped capsular protrusion, whose elastic recoil (9) constricts the incision edges, creating an irreducible incarcerated capsular hernia (see Figure 4). This pathological situation is often exacerbated by concurrent vitreous incarceration at the incision site.

Based on its underlying mechanism, we summarized the possible main challenges in managing ICBH as follows: (1) Persistent capsular herniation under high IOP makes manual reduction difficult; (2) The capsular bag, excessively distended by retained OVD, herniate through the main incision, compressing the incision edges and perpetuating the incarceration; (3) Increased pressure within the herniated portion of the capsular bag, combined with the intrinsic elasticity of the capsule, may progressively thin the herniated capsule, making it more susceptible to rupture during manipulation; (4) Concurrent vitreous prolapse and incarceration further complicate surgical maneuvers; and (5) The herniated capsule significantly obstructs visibility at the incision, impairing intraoperative visualization. Critically, forceful manual reduction of the incarcerated capsule can induce capsular rupture.

**Figure 5:**
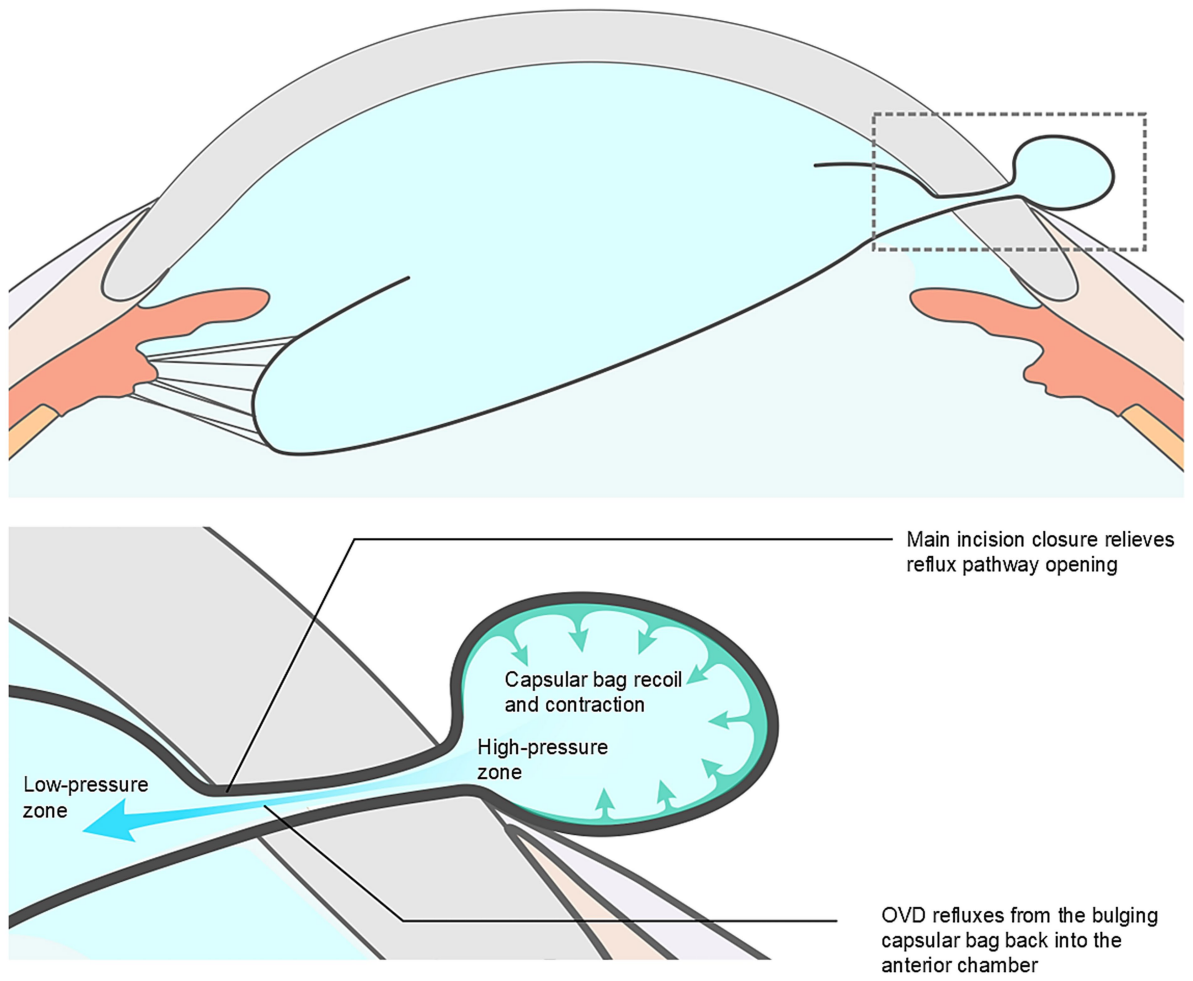
Proposed mechanism of capsular bag repositioning. A favorable pressure gradient is created between the distended capsular bag and the anterior chamber, facilitating ophthalmic viscosurgical device (OVD) reflux into the anterior chamber and resulting in capsular decompression, thereby disrupting the mushroom-shaped configuration.

Intraoperative prolapse of the iris or vitreous through the main incision is a recognized complication during subluxated lens surgery, typically managed through strategies such as IOP reduction, tissue repositioning, and anterior vitrectomy. However, the management of ICBH presents distinct challenges due to the unique anatomical and biomechanical properties of the capsular bag. Unlike the iris, which is elastic and generally amenable to repositioning, the capsular bag is structurally fragile and suspended by delicate zonular fibers. The herniated segment often becomes internally pressurized, particularly after OVD injection, resulting in a distended and non-reducible configuration once it protrudes through the tight corneal incision. This condition forms a fixed incarceration that is more difficult to reposition than iris prolapse. Furthermore, in contrast to vitreous prolapse, which can be definitively treated with vitrectomy, the capsular bag must be preserved intact for successful in-the-bag IOL implantation, precluding excisional approaches and necessitating precise and atraumatic reduction techniques. As a possible strategy to address these challenges, we propose a standardized stepwise surgical approach. (1) IOP reduction: A blunt cannula is inserted through the corneal side-port incision to gently depress the posterior lip of the incision, allowing controlled egress of OVD. Given the small incision size and the cohesive properties of the OVD (10), this maneuver may require repeated gentle compressions to achieve sufficient IOP reduction. (2) Relief of incision incarceration: The same cannula is advanced through the side-port toward the posterior lip of the main corneal incision to carefully release the constriction on the herniated portion of the capsular bag. (3) Capsular decompression: A favorable pressure gradient is established between the distended capsular bag and the anterior chamber, facilitating OVD reflux into the anterior chamber and thereby disrupting the mushroom-shaped configuration (see Figure 5). (4) Gradual capsular repositioning: With successive pressure equalizations, gentle sweeping motions are applied to progressively reposition the herniated capsule into the anterior chamber, minimizing tangential stress and reducing the risk of capsular tears. (5) Vitreous clearance: Meticulous anterior vitrectomy is performed to remove any prolapsed vitreous strands (11). This management strategy effectively disrupts the vicious cycle of elevated IOP, capsular bag herniation with incarceration, and recurrent herniation.

The following preventive measures are proposed as potential strategies to reduce the risk of ICBH. (1) Comprehensive preoperative imaging evaluations to precisely determine zonular dialysis location and extent; (2) Strategic placement of the main corneal incision away from areas of zonular compromise; (3) Strict control of intraoperative IOP by carefully adjusting infusion parameters and limiting OVD injection volumes; and (4) Avoidance of sudden anterior chamber decompression by constructing appropriately sized corneal incisions and employing meticulous instrument handling techniques.

In all cases of this study, despite the occurrence of ICBH, successful preservation of the capsular bag was achieved, allowing for in-the-bag IOL implantation. Postoperative follow-up showed improved visual acuity compared to baseline, with stable IOP and well-centered IOLs in all cases. Case 2 developed cystoid macular edema postoperatively, which may be attributed to postoperative inflammatory response, vitreous traction, and the use of a CTR (12). Choroidal detachment occurred in Case 3 one month after surgery, consistent with the known risk associated with nanophthalmos (13).

In summary, ICBH was an uncommon but clinically important complication during cataract surgery for lens subluxation. ICBH arises from a combination of zonular weakness, corneal incision location, sudden IOP fluctuations, incision-related forces, the fluid dynamics of OVDs, and the biomechanical properties of the capsular bag. A stepwise approach incorporating controlled IOP reduction, capsular bag decompression, repositioning of the prolapsed capsule bag, and vitreous clearance resulted in favorable outcomes. Preventive strategies are presumed to include preoperative zonular assessment, strategic incision placement, and meticulous intraoperative pressure control. Given the rarity of ICBH and the small sample size, this study did not include inferential statistical analysis. Future studies with larger cohorts are needed to confirm these observations.

## Data Availability

The original contributions presented in the study are included in the article/Supplementary material, further inquiries can be directed to the corresponding author.
